# Evidence of behaviour change during an Ebola virus disease outbreak, Sierra Leone

**DOI:** 10.2471/BLT.19.245803

**Published:** 2020-03-26

**Authors:** Mohamed F Jalloh, Paul Sengeh, Rebecca E Bunnell, Mohammad B Jalloh, Roeland Monasch, Wenshu Li, Jonathan Mermin, Nickolas DeLuca, Vance Brown, Sophia A Nur, Euna M August, Ray L Ransom, Apophia Namageyo-Funa, Sara A Clements, Meredith Dyson, Kathy Hageman, Samuel Abu Pratt, Azizeh Nuriddin, Dianna D Carroll, Nicole Hawk, Craig Manning, Sara Hersey, Barbara J Marston, Peter H Kilmarx, Lansana Conteh, Anna Mia Ekström, Zangin Zeebari, John T Redd, Helena Nordenstedt, Oliver Morgan

**Affiliations:** a Department of Global Public Health, Karolinska Institutet, Tomtebodavägen 18B, 17165 Solna, Sweden.; b FOCUS 1000, Freetown, Sierra Leone.; c Centers for Disease Control and Prevention, Atlanta, United States of America (USA).; d United Nations Children’s Fund, Freetown, Sierra Leone.; e Catholic Relief Services, Freetown, Sierra Leone.; f Sierra Leone Ministry of Health and Sanitation, Freetown, Sierra Leone.

## Abstract

**Objective:**

To evaluate changes in Ebola-related knowledge, attitudes and prevention practices during the Sierra Leone outbreak between 2014 and 2015.

**Methods:**

Four cluster surveys were conducted: two before the outbreak peak (3499 participants) and two after (7104 participants). We assessed the effect of temporal and geographical factors on 16 knowledge, attitude and practice outcomes.

**Findings:**

Fourteen of 16 knowledge, attitude and prevention practice outcomes improved across all regions from before to after the outbreak peak. The proportion of respondents willing to: (i) welcome Ebola survivors back into the community increased from 60.0% to 89.4% (adjusted odds ratio, aOR: 6.0; 95% confidence interval, CI: 3.9–9.1); and (ii) wait for a burial team following a relative’s death increased from 86.0% to 95.9% (aOR: 4.4; 95% CI: 3.2–6.0). The proportion avoiding unsafe traditional burials increased from 27.3% to 48.2% (aOR: 3.1; 95% CI: 2.4–4.2) and the proportion believing spiritual healers can treat Ebola decreased from 15.9% to 5.0% (aOR: 0.2; 95% CI: 0.1–0.3). The likelihood respondents would wait for burial teams increased more in high-transmission (aOR: 6.2; 95% CI: 4.2–9.1) than low-transmission (aOR: 2.3; 95% CI: 1.4–3.8) regions. Self-reported avoidance of physical contact with corpses increased in high but not low-transmission regions, aOR: 1.9 (95% CI: 1.4–2.5) and aOR: 0.8 (95% CI: 0.6–1.2), respectively.

**Conclusion:**

Ebola knowledge, attitudes and prevention practices improved during the Sierra Leone outbreak, especially in high-transmission regions. Behaviourally-targeted community engagement should be prioritized early during outbreaks.

## Résumé

**Objectif:**

Évaluer l'évolution des connaissances, attitudes et techniques de prévention en matière d'Ebola durant l'épidémie qui a touché la Sierra Leone entre 2014 et 2015.

**Méthodes:**

Quatre enquêtes en grappes ont été menées: deux avant le pic de l'épidémie (3499 participants) et deux après (7104 participants). Nous avons mesuré l'impact des facteurs géographiques et temporels sur 16 résultats liés aux connaissances, aux attitudes et aux techniques de prévention.

**Résultats:**

Quatorze des seize résultats liés aux connaissances, aux attitudes et aux techniques de prévention ont progressé dans toutes les régions entre la période avant le pic et celle après le pic. La proportion de répondants disposés à: (i) accueillir les survivants à Ebola de retour dans leur communauté est passée de 60,0% à 89,4% (odds ratio ajusté, ORA: 6,0; intervalle de confiance de 95%, IC: 3,9–9,1); et (ii) attendre l'équipe d'inhumation après la mort d'un proche a également augmenté, passant de 86,0% à 95,9% (ORA: 4,4; IC de 95%: 3,2–6,0). La proportion de répondants ayant abandonné la pratique risquée des funérailles traditionnelles est passée de 27,3% à 48,2% (ORA: 3,1; IC de 95%: 2,4–4,2), tandis que celle convaincue de l'efficacité des guérisseurs spirituels pour traiter Ebola a diminué, passant de 15,9% à 5,0% (ORA: 0,2; IC de 95%: 0,1-0,3). La probabilité que les répondants attendent les équipes d'inhumation a augmenté dans les régions à haut risque de transmission (ORA: 6,2; IC de 95%: 4,2–9,1), plus que dans les régions à faible risque de transmission (ORA: 2,3; IC de 95%: 1,4–3,8). Les répondants déclarent avoir davantage évité tout contact physique avec les corps dans les régions à haut risque de transmission, mais pas dans les régions à faible risque de transmission (ORA: 1,9; IC de 95%: 1,4–2,5 et ORA: 0,8; IC de 95%: 0,6–1,2).

**Conclusion:**

Les connaissances, attitudes et techniques de prévention en matière d'Ebola ont évolué durant l'épidémie qui a touché la Sierra Leone, surtout dans les régions à haut risque de transmission. Il faut privilégier l'engagement communautaire axé sur le comportement dès les premiers stades de l'épidémie.

## Resumen

**Objetivo:**

Evaluar los cambios en los conocimientos, las actitudes y las prácticas de prevención relacionadas con el ébola durante el brote ocurrido en Sierra Leona entre 2014 y 2015.

**Métodos:**

Se realizaron cuatro encuestas por conglomerados: dos antes del pico del brote (3499 participantes) y dos después (7104 participantes). Se evaluó el efecto de los factores temporales y geográficos en 16 resultados de conocimientos, actitudes y prácticas de prevención.

**Resultados:**

14 de 16 resultados de conocimientos, actitudes y prácticas de prevención mejoraron en todas las regiones desde antes hasta después del pico del brote. El porcentaje de los encuestados dispuestos a: (i) acoger a los supervivientes del ébola en la comunidad aumentó del 60,0 % al 89,4 % (proporción de probabilidad ajustada, aOR: 6,0; intervalo de confianza del 95 %, IC: 3,9-9,1); y (ii) esperar al servicio funerario tras la muerte de un familiar aumentó del 86,0 % al 95,9 % (aOR: 4,4; IC del 95 %: 3,2-6,0). El porcentaje de personas que evitaron los entierros tradicionales inseguros aumentó del 27,3 % al 48,2 % (aOR: 3,1; IC del 95 %: 2,4-4,2) y el porcentaje de personas que creen que los sanadores espirituales pueden tratar el ébola disminuyó del 15,9 % al 5,0 % (aOR: 0,2; IC del 95 %: 0,1-0,3). La probabilidad de que los encuestados esperan a los servicios funerarios aumentó más en las regiones de alta transmisión (aOR: 6,2; IC del 95 %: 4,2-9,1) que en las de baja transmisión (aOR: 2,3; IC del 95 %: 1,4-3,8). Según los reportes de los mismos encuestados, se evitó más el contacto físico con los cadáveres en las regiones de alta pero no de baja transmisión, aOR: 1,9 (IC del 95 %: 1,4-2,5) y aOR: 0,8 (IC del 95 %: 0,6-1,2), respectivamente.

**Conclusión:**

Los conocimientos, las actitudes y las prácticas de prevención del ébola mejoraron durante el brote en Sierra Leona, en especial en las regiones de alta transmisión. Se debe dar prioridad a la participación de la comunidad orientada al comportamiento en las primeras etapas de los brotes.

## ملخص

**الغرض:**

تقييم التغييرات في المعارف والمواقف وممارسات الوقاية المتعلقة بفيروس إيبولا أثناء تفشيه في سيراليون بين عامي 2014 و2015.

**الطريقة:**

قم بإجراء أربعة مسوحات مجمعة: اثنين قبل ذروة التفشي (بمشاركة 3499 شخصاً)، واثنين بعدها (بمشاركة 7104 شخصاً). قمنا بتقييم تأثير العوامل الزمنية والجغرافية على 16 من النتائج المعرفية ونتائج المواقف والممارسة.

**النتائج:**

تحسنت 14 من 16 نتيجةً للمعرفة والمواقف وممارسات الوقاية عبر كل المناطق، سواء من قبل أو بعد ذروة التفشي. نسبة المستجيبين الراغبين في: (1) الترحيب بعودة الناجين من الإصابة بإيبولا إلى المجتمع، ارتفعت من 60.0% إلى 89.4% (نسبة الاحتمالات المعدلة: 6.0؛ فاصل الثقة 95%: 3.9 إلى 9.1)؛ و(2) ارتفعت نسبة انتظار فريق الدفن بعد وفاة أحد الأقارب، من 86.0% إلى 95.9% (نسبة الاحتمالات المعدلة: 4.4؛ ‏بفاصل ثقة 95%: من 3.2 إلى 6.0). ارتفعت نسبة تجنب عمليات الدفن التقليدي غير الآمن من 27.3% إلى 48.2% (نسبة الاحتمالات المعدلة: 3.1؛ ‏بفاصل ثقة 95%: 2.4 إلى 4.2)، وانخفضت نسبة المصدقين بالمعالجين الروحيين الذين يمكنهم علاج إيبولا من 15.9% إلى 5.0% (نسبة الاحتمالات المعدلة: 0.2؛ ‏بفاصل ثقة 95%: من 0.1 إلى 0.3). ارتفعت نسبة احتمال انتظار المستجيبين لفرق الدفن في مناطق الانتشار السريع (نسبة الاحتمالات المعدلة: 6.2؛ ‏بفاصل ثقة 95%: 4.2 إلى 9.1) عن مناطق الانتشار المنخفض (نسبة الاحتمالات المعدلة: 2.3؛ ‏بفاصل ثقة 95%: 1.4 إلى 3.8). ازدادت نسبة الإبلاغ الذاتي عن تجنب التلامس الجسدي مع الجثث في المناطق ذات الانتشار المرتفع، وليس الانتشار المنخفض، نسبة الاحتمالات المعدلة: 1.9 (فاصل الثقة 95%: 1.4 إلى 2.5) ونسبة الاحتمالات المعدلة: 0.8 (فاصل الثقة 95%: 0.6 إلى 1.2)، على الترتيب.

**الاستنتاج:**

شهدت المعارف والمواقف وممارسات الوقاية المتعلقة بفيروس إيبولا، تحسناً أثناء تفشيه في سيراليون، وخاصة في المناطق ذات الانتشار المرتفع. يجب توجيه الأولوية لإشراك المجتمع المستهدف سلوكياً في وقت مبكر أثناء حالات التفشي.

## 摘要

**目的:**

旨在评估 2014 年至 2015 年塞拉利昂疫情期间与埃博拉相关知识、态度和预防措施的变化。

**方法:**

进行四次群组调查：疫情高峰期前两次（3499 位参与者），疫情高峰期后两次（7104 位参与者）。我们评估了时间和地理因素对 16 个知识、态度和措施结果的影响。

**结果:**

16 个知识、态度和预防措施结果中有 14 个在各地区爆发高峰期前至爆发高峰期后都有所改善。受访者中，愿意：(i) 欢迎埃博拉幸存者重返社区的受访者比例从 60.0% 增加到 89.4%（调整后比值，aOR：6.0；95% 置信区间，CI：3.9–9.1）；和 (ii) 亲属死亡后等待埋葬队的受访者比例从 86.0% 增加到 95.9% (aOR：4.4；95% CI: 3.2-6.0）。避免不安全的传统葬礼的比例从 27.3% 增加到 48.2% (aOR：3.1；95% CI: 2.4–4.2) ，相信精神治疗师可以治疗埃博拉的比例从 15.9% 降低到 5.0% (aOR：0.2；95% CI: 0.1-0.3）。受访者等待埋葬队的可能性在高传播地区 (aOR：6.2；95% CI: 4.2–9.1) 比低传播地区 (aOR: 2.3；95% CI: 1.4-3.8) 更高。高传播地区受访者自述避免与尸体发生实际接触的情况有所增加，而低传播地区无此趋势，分别为 aOR：1.9 (95% CI: 1.4–2.5) 和 aOR: 0.8 (95% CI: 0.6–1.2)。

**结论:**

在塞拉利昂疫情期间，特别是在高传播地区，埃博拉知识、态度和预防措施已得到改善。在行为上，具有针对性的社区参与应在疫情爆发早期被确定优先事项。

## Резюме

**Цель:**

Оценить изменения в наших знаниях о лихорадке Эбола, в поведении и в профилактических действиях во время вспышки заболевания в Сьерра-Леоне на протяжении 2014–2015 гг.

**Методы:**

Было проведено четыре кластерных опроса: два до пика заболевания (3499 участников) и два после (7104 участника). Мы оценили воздействие временного и географического факторов на 16 показателей, связанных со знаниями, поведением и действиями.

**Результаты:**

Четырнадцать из 16 показателей в области знаний, поведения и профилактических действий улучшились по всем регионам после вспышки по сравнению с предшествующей ситуацией. Доля респондентов: (i) которые не возражали против возвращения выживших больных в общину, выросла с 60,0 до 89,4% (скорректированное отношение шансов, сОШ: 6,0; 95%-й ДИ: 3,9–9,1); (ii) которые дожидались прибытия похоронной команды после смерти родственника, выросла с 86,0 до 95,9% (сОШ: 4,4; 95%-й ДИ: 3,2–6,0). Доля тех, кто избегал небезопасных традиционных похоронных практик, выросла с 27,3 до 48,2% (сОШ: 3,1; 95%-й ДИ: 2,4–4,2), и доля тех, кто считал, что духовные целители могут вылечить лихорадку Эбола, снизилась с 15,9 до 5,0% (сОШ: 0,2; 95%-й ДИ: 0,1–0,3). Вероятность того, что респонденты будут ждать прибытия похоронной команды, сильнее выросла в зонах с высоким уровнем передачи инфекции (сОШ: 6,2; 95%-й ДИ: 4,2–9,1), чем в зоне с низким уровнем передачи (сОШ: 2,3; 95%-й ДИ: 1,4–3,8). Частота сообщений о том, что респондент избегает физического контакта с трупами, выросла в зонах высокой передачи инфекции, но не в зонах ее низкой передачи, сОШ 1,9 (95%-й ДИ: 1,4–2,5) и сОШ 0,8 (95%-й ДИ: 0,6–1,2) соответственно.

**Вывод:**

Знания, поведение и действия, предотвращающие распространение лихорадки Эбола, в ходе вспышки в Сьерра-Леоне улучшились, особенно в регионах с высоким уровнем передачи инфекции. На ранних этапах развития вспышки следует на первое место поставить участие общины и организацию правильного ее поведения.

## Introduction

The 2013–2016 Ebola virus disease outbreak in West Africa mostly affected Guinea, Liberia and Sierra Leone. In Sierra Leone, over 14 000 cases of Ebola and about 4000 deaths were confirmed between May 2014 and January 2016, which made it the largest documented outbreak of the disease to date.^1^ Governments and their partner organizations rallied to strengthen their capacity to respond by: (i) identifying and isolating suspected cases; (ii) implementing safe burials by specialized teams; and (iii) instituting stringent infection prevention and control measures at health facilities.^2^ The modification of traditional burial practices, which involve contact with corpses, and caregiving practices, which involve physical contact with patients, were critical for outbreak control.^3^,^4^

The Government of Sierra Leone established a social mobilization pillar less than a month after the outbreak was declared. Radio provided the main mode of communicating with the public about Ebola during the early phase of the response because of its advantages over other communication methods: it is cheaper, it has a national reach and messages can be delivered rapidly.^5^ As the outbreak progressed, social mobilization efforts shifted from one-way communication to structured community engagement.^6^,^7^ Over 6000 religious leaders were engaged to promote safe burials and 2500 full-time community mobilizers facilitated community-led action plans.^7^,^8^

Mathematical modelling has indicated that improvements in behaviour contribute to controlling Ebola outbreaks.^3^,^9^,^10^ One model demonstrated that Ebola treatment-seeking approximately doubled during the outbreak in Lofa County, Liberia; another revealed that improved public education contributed to better prevention practices in South Sudan, which resulted in fewer Ebola cases.^11^ However, an inherent limitation of these mathematical models is that they were not based on actual behavioural data. In addition, individual surveys of Ebola knowledge, attitudes and prevention practices conducted during the West Africa outbreak revealed that good knowledge of the disease and high uptake of prevention behaviours existed alongside prevailing misconceptions.^12^–^15^ Prevention practices may have been influenced by intrinsic and extrinsic factors.^9^,^16^ Intrinsic factors include lived experiences (e.g. observing the death of family members who attend traditional funerals) and extrinsic factors include planned social mobilization and community engagement interventions. However, there remained a lack of information on the magnitude of the changes in the public’s knowledge and practices that took place as outbreaks progressed.

The aim of our study was to examine trends in knowledge about the Ebola virus disease, acceptance of safe burial practices, attitudes towards Ebola survivors and the uptake of prevention practices during the Ebola outbreak in Sierra Leone between 2014 and 2015. In addition, we reflect on the key lessons learnt while implementing surveys during an unprecedented disease outbreak, which we hope will inform real-time behavioural assessments during other similar outbreaks.

## Methods

We conducted four cross-sectional, household surveys of Ebola knowledge, attitudes and prevention practices in August 2014, October 2014, December 2014 and July 2015, respectively, during the Sierra Leone outbreak. The first survey covered 9 of the 14 administrative districts; these districts were selected because disease transmission was occurring at that time.^5^ The subsequent three surveys covered all 14 districts. For each survey, we used multistage, cluster sampling procedures, with the 2004 Sierra Leone census list of enumeration areas serving as a sampling frame for the random selection of enumeration areas (i.e. clusters) within districts.^17^ A systematic, random sampling technique was used to select households within each cluster.^18^ For each cluster, a sampling interval (i.e. the number of households in the cluster divided by the number of households to be sampled) was calculated in advance for use by the data collection team. The team randomly selected a household located in the centre of the cluster as the starting point for each survey and additional households were then selected using the sampling interval until the desired sample of the cluster had been reached.

For each household, data collectors selected two eligible individuals to interview. The first was always the household head because of his or her influence on household decisions and practices. As the cultural norm in Sierra Leone is that household heads are usually older men, the second interviewee randomly selected from the household was either an adult woman aged 25 years or older or a young person aged 15 to 24 years. To obtain the district-level estimates needed to inform and guide targeted social mobilization activities in active Ebola transmission areas, we oversampled Western Area Urban, Western Area Rural and Port Loko districts in December 2014 and July 2015, Kailahun district in December 2014 and Kambia district in July 2015. Details of the social mobilization activities carried out at different stages of the outbreak are available from the corresponding author on request.

### Questionnaire

Details of the survey questionnaire are presented in Table 1. The survey included questions on 16 outcome measures across five domains, which were informed by the literature on other communicable diseases:^19^–^22^ (i) knowledge; (ii) misconceptions; (iii) social acceptance of survivors; (iv) acceptance of safe burial practices; and (v) self-reported prevention practices. Most items required a close-ended response of “yes,” “no” or “don’t know.” For items on self-reported prevention practices, however, an open-ended response was sought to enable participants to give several unprompted responses. Although the questionnaire included pre-coded response categories to capture open-ended responses on prevention practices, participants were not aware of these categories.

**Table 1:** Questionnaire, Ebola knowledge, attitude and prevention practice surveys, Sierra Leone, 2014–2015

Domain and measure	Item	Response options	Format
**Knowledge**			
1. Ebola is preventable by avoiding contact with a corpse	Can I prevent myself from getting Ebola by avoiding funeral or burial rituals that require handling the body of someone who has died from Ebola?	Yes, no or don’t know/not sure	Prompted, single response only
2. Early medical care of Ebola increases the chance of survival	If a person has Ebola has he/she a higher chance of survival if he/she goes immediately to a health facility?	Yes, no or don’t know/not sure	Prompted, single response only
3. Early medical care of Ebola reduces household transmission	If a person with Ebola goes immediately to a health facility will he/she reduce the chance of spreading it to their family or people living with them?	Yes, no or don’t know/not sure	Prompted, single response only
**Misconception**			
4. Bathing with salt and hot water prevents Ebola	Can I prevent myself from getting Ebola by bathing with salt and hot water?	Yes, no or don’t know/not sure	Prompted, single response only
5. Spiritual healers can successfully treat Ebola	Do you believe that spiritual healers can treat Ebola successfully?	Yes, no or don’t know/not sure	Prompted, single response only
6. Traditional healers can successfully treat Ebola	Do you believe that traditional healers can treat Ebola successfully?	Yes, no or don’t know/not sure	Prompted, single response only
**Social acceptance of survivors**			
7. Would welcome back Ebola survivor into the community	Would you welcome someone back into your community/neighbourhood after he/she has recovered from Ebola?	Yes, no or don’t know/not sure	Prompted, single response only
8. Would buy fresh vegetables from Ebola survivor shopkeeper	Would you buy fresh vegetables from a shopkeeper who survived Ebola and has a certificate from a government health facility stating he/she is now Ebola-free?	Yes, no or don’t know/not sure	Prompted, single response only
9. Ebola survivor student does not put class at risk of Ebola	Do you think that a school pupil who has survived Ebola and has a certificate from a government health facility stating he/she is Ebola-free puts other pupils in their class at risk of infection?	Yes, no or don’t know/not sure	Prompted, single response only
**Acceptance of safe burial practices**			
10. Would avoid touching or washing a corpse	If a family member became sick and died tomorrow, would you touch or wash the dead body?	Yes, no or don’t know/not sure	Prompted, single response only
11. Would wait for the Ebola burial team to bury the body	If a family member became sick and died tomorrow, would you wait for the burial team to bury the body?	Yes, no or don’t know/not sure	Prompted, single response only
12. Would accept safe alternatives to traditional burial rituals	If a family member died, would you accept alternatives to a traditional funeral/burial that would NOT involve touching or washing the dead body?	Yes, no or don’t know/not sure	Prompted, single response only
**Self-reported prevention practices^a^**			
13. Uptake of any Ebola prevention practice	Since you heard of Ebola, have you taken any action to avoid being infected?	Open-ended	Unprompted, multiple responses allowed
14. Wash hands with soap and water more often	In what ways have you changed your behaviour or taken actions to avoid being infected? (Only asked if the respondent answered “yes” to question 13)	Open-ended	Unprompted, multiple responses allowed
15. Avoid physical contact with suspected Ebola patients	In what ways have you changed your behaviour or taken actions to avoid being infected? (Only asked if the respondent answered “yes” to question 13)	Open-ended	Unprompted, multiple responses allowed
16. Avoid burials that involve contact with a corpse	In what ways have you changed your behaviour or taken actions to avoid being infected? (Only asked if the respondent answered “yes” to question 13)	Open-ended	Unprompted, multiple responses allowed

^a^ Other pre-coded response categories for prevention practices included: (i) I wash my hands with just water more often; (ii) I clean my hands with other disinfectants more often; (iii) I try to avoid crowded places; (iv) I drink Bittercola; (v) I drink a lot of water or juice; (vi) I drink traditional herbs; (vii) I take antibiotics; (viii) I wear gloves; (ix) I wash with salt and hot water; (x) I use a condom when having sex with someone who has survived Ebola; (xi) I always use a condom when having sex; (xii) I don’t know / am not sure; and (xiii) other unprompted responses.

For each survey, questionnaires were tested in a pilot study using convenience samples that were excluded from the final sample. We subsequently revised the questionnaires to improve the sequencing of items and to take account of local terminology. Respective questionnaires were orally translated into Krio (the most widely spoken local language) and other local languages during the training of data collectors. The data collectors mostly interviewed in Krio with oral translation into other local languages as needed. A nongovernmental organization, FOCUS 1000, implemented data collection. The first survey used a paper-based questionnaire, whereas subsequent surveys were administered using Android tablet computers, which were loaded with surveys containing standardized data elements and skip patterns developed using an Open Data Kit software application.^23^

### Statistical analysis

**Fig. 1:**
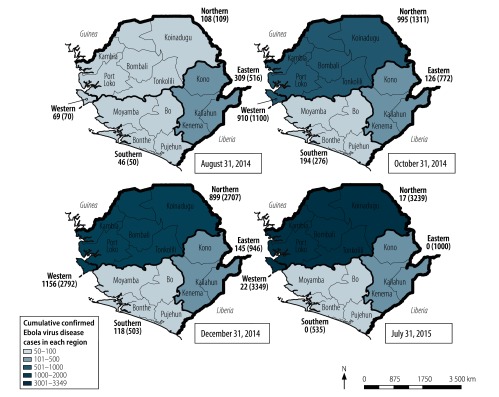
New and cumulative Ebola virus disease cases at the time of the four surveys of Ebola knowledge, attitudes and prevention practices, by region, Sierra Leone, 2014–2015

Notes: For each survey date, the illustration shows the number of Ebola virus disease cases confirmed in the previous 42 days in each of the four regions, with the cumulative total in parentheses. The western region includes two districts: Western Area Urban and Western Area Rural districts. Across the four surveys, 258 enumeration areas (i.e. clusters) were sampled from a total of around 10 000 enumeration areas in the country. As 24 enumeration areas were sampled more than once during randomization, 234 unique clusters were visited in the four data collection rounds, which represent approximately 2.5% of the national number of enumeration areas in the 2004 census. On average, 100 households (range: 50–120) were selected in each enumeration area.

All four surveys were designed to produce national and regional estimates at the 95% confidence level within a 2.5% margin of error for national estimates and a 3.5% margin of error for regional estimates on the assumption that 50% of respondents would know three Ebola prevention or treatment measures. Data from the four surveys were pooled into a combined data set and analysed using Stata/SE version 15 (StataCorp LLC, Cary, United States of America). The svy command in Stata was used to adjust for the effect of the multistage sampling approach on the calculation of point estimates and their standard errors.^24^ As the peak of the outbreak in Sierra Leone occurred in November 2014, the surveys conducted in August 2014 and October 2014 were regarded as taking place before the peak and the surveys in December 2014 and July 2015 were regarded as taking place after the peak. The four geographical regions of the country (i.e. eastern, western, northern and southern) were dichotomized into low- and high-transmission regions according to the cumulative number of confirmed Ebola cases recorded by the World Health Organization (WHO) after the outbreak.^1^ Western and northern regions were categorized as high-transmission (i.e. over 3000 cases per region cumulatively) and eastern and southern regions were categorized as low-transmission (i.e. 1000 or fewer cases per region cumulatively; Fig. 1). The high- and low-transmission regions corresponded to the high- and low-mortality regions. In trying to understand the potential effect of changes in the population’s knowledge, attitudes and prevention practices on containing the outbreak, we chose to focus on differences between these high- and low-transmission regions. 

The number and proportion of survey participants who gave the desired responses to the survey questions before and after the outbreak peak are presented in the tables. Differences in the odds of individual knowledge, attitude and practice outcomes between before and after the outbreak peak were analysed using multilevel logistic regression models with random intercepts to account for the random effects of clusters. Models were adjusted for the type of region (high or low transmission) and the respondents’ sex (male or female), age (15 to 24 years of age or  25 years of age or older), educational level (no education, primary, secondary or higher) and religious affiliation (Muslim, Christian or other). In addition, we used a multilevel model to account for the random effects of the geographical clustering of respondents over time, this model was adjusted for demographic variations. Then we added an interaction term to the models to estimate the combined effect of temporal and geographical interactions on knowledge, attitude and practice outcomes. We set the level of significance at 0.05 in all models.

## Results

In total, 10 603 respondents consented to participating in the surveys: 1413 in August 2014, 2086 in October 2014, 3540 in December 2014 and 3564 in July 2015. The overall response rate was 98.5% (10 603/10 760). Furthermore, 49.9% (5289/10 591) were female, 33.5% (3531/10 554) had no formal education, 67.3% (7127/10 583) identified as Muslim, 20.7% (2181/10 535) were farmers and 23.1% (2434/10 535) were students (Table 2).

**Table 2:** Respondents characteristics of the Ebola knowledge, attitude and prevention practice surveys, Sierra Leone, 2014–2015

Respondents’ characteristics	Number of survey respondents (% of observations)^a^				
	Survey date				Total (*n* = 10 603)
	August 2014 (*n* = 1413)	October 2014 (*n* = 2086)	December 2014 (*n* = 3540)	July 2015 (*n* = 3564)	
**Region of residence**					
Western	431 (30.5)	522 (25.0)	812 (22.9)	798 (22.4)	2563 (24.2)
Northern	435 (30.8)	633 (30.4)	1247 (35.2)	1740 (48.8)	4055 (38.2)
Eastern	269 (19.0)	420 (20.1)	919 (26.0)	471 (13.2)	2079 (19.6)
Southern	278 (19.7)	511 (24.5)	562 (15.9)	555 (15.6)	1906 (18.0)
**Sex**					
Male	749 (53.4)	970 (46.6)	1809 (51.1)	1774 (49.8)	5302 (50.1)
Female	655 (46.6)	1113 (53.4)	1731 (48.9)	1790 (50.2)	5289 (49.9)
**Age, years**					
15–24	511 (36.7)	741 (35.6)	1177 (33.3)	1203 (33.8)	3632 (34.4)
≥ 25	880 (63.3)	1340 (64.4)	2362 (67.7)	2362 (66.2)	6942 (66.6)
**Education**					
None	360 (26.0)	553 (26.7)	1194 (33.8)	1424 (40.0)	3531 (33.5)
Some primary	188 (13.5)	360 (17.4)	677 (19.1)	739 (20.8)	1964 (18.6)
Secondary or higher	840 (60.5)	1157 (55.9)	1668 (47.1)	1394 (39.2)	5059 (47.9)
**Religion**					
Islam	901 (64.2)	1342 (64.5)	2335 (66.0)	2459 (71.5)	7127 (67.3)
Christianity	501 (35.7)	736 (35.4)	1200 (33.9)	1015 (28.5)	3452 (33.6)
Other	1 (0.1)	1 (0.1)	2 (0.1)	0 (0.0)	4 (0.1)
**Occupation**					
Farmer	136 (9.7)	242 (11.6)	891 (25.2)	912 (25.6)	2181 (20.7)
Small trader	272 (19.3)	395 (19.0)	614 (17.3)	735 (20.6)	2016 (19.0)
Student	360 (25.5)	556 (26.7)	795 (22.5)	723 (20.3)	2434 (23.1)
Private business employee	93 (6.6)	170 (8.2)	286 (8.1)	268 (7.5)	817 (7.7)
Teacher	99 (7.0)	154 (7.4)	187 (5.3)	144 (4.0)	584 (5.5)
Health worker	26 (1.8)	42 (2.0)	40 (1.1)	32 (0.9)	140 (1.3)
Other government worker	86 (6.1)	92 (4.4)	153 (4.3)	98 (2.8)	429 (4.1)
Driver	12 (0.9)	34 (1.6)	51 (1.4)	47 (1.3)	144 (1.4)
Bike rider	21 (1.5)	20 (1.0)	50 (1.4)	58 (1.6)	149 (1.4)
Skilled labourer	56 (4.0)	104 (5.0)	111 (3.1)	113 (3.2)	384 (3.6)
Retired	0 (0.0)	0 (0.0)	0 (0.0)	51 (1.4)	51 (0.5)
Unemployed	208 (14.8)	268 (12.9)	356 (10.0)	351 (9.9)	1183 (11.2)
Other	0 (0.0)	0 (0.0)	0 (0.0)	23 (0.7)	23 (0.2)

^a^ The total number of missing values for all demographic characteristics was less than 1% of all responses: there were 12 missing responses for sex, 29 for age, 49 for education, 20 for religion and 68 for occupation.

Between the early phase of the outbreak in August 2014 and near the peak in October 2014, knowledge of the Ebola virus disease became more common and social acceptance of Ebola survivors increased markedly. Between October and December 2014, acceptance of safe burials increased notably, as did most self-reported prevention practices (Table 3). There were significant improvements from before to after the outbreak peak in 14 of the 16 knowledge, attitude and practice outcomes (Table 4; available at: http://www.who.int/bulletin/volumes/98/5/19-245803). One of the two measures that did not improve was knowledge that early medical care of Ebola virus disease reduces the risk of household transmission: 92.6% (3226/3483) of respondents reported this knowledge before the peak compared with 92.3% (6552/7097) after. In addition, 96.4% (3366/3493) of respondents reported they had taken one or more actions to prevent Ebola virus disease before the peak compared with 97.3% (6894/7104) after.

**Table 3:** Surveys of Ebola knowledge, attitudes and prevention practices during an outbreak, Sierra Leone, 2014–2015

Ebola knowledge, attitude or prevention practice	Respondents giving a positive response, by survey date							
	August 2014 (*n* = 1413)		October 2014 (*n* = 2086)		December 2014 (*n* = 3540)		July 2015 (*n* = 3564)	
	No.^a^	% (95% CI)^b^	No.^c^	% (95% CI)^b^	No.^d^	% (95% CI)^b^	No.^e^	% (95% CI)^b^
**Knowledge**								
1. Ebola is preventable by avoiding contact with a corpse	1182	84.7 (77.9–89.7)	1959	94.3 (92.4–95.8)	3414	96.4 (95.3–97.4)	3327	93.4 (91.6–94.9)
2. Early medical care of Ebola increases the chance of survival	1254	90.3 (86.7–93.0)	1938	93.3 (91.4–94.8)	3372	95.4 (94.0–96.4)	3419	96.0 (94.9–96.9)
3. Early medical care of Ebola reduces household transmission	1284	91.3 (86.8–94.4)	1942	93.5 (91.9–94.8)	3258	92.1 (90.1–93.8)	3294	92.5 (90.9–93.9)
**Misconception**								
4. Bathing with salt and hot water prevents Ebola	571	41.6 (37.4–46.0)	717	34.5 (31.5–37.5)	1117	31.6 (28.0–35.4)	534	15.0 (12.6–17.8)
5. Spiritual healers can successfully treat Ebola	275	19.6 (14.8–25.6)	278	13.4 (10.8–16.4)	207	5.8 (4.6–7.4)	145	4.1 (2.8–5.8)
6. Traditional healers can successfully treat Ebola	80	5.7 (4.3–7.5)	66	3.2 (2.4–4.1)	66	1.9 (1.4–2.5)	46	1.3 (0.8–1.9)
**Social acceptance of survivors**								
7. Would welcome back Ebola survivor into the community	312	22.4 (17.2–29.0)	1772	85.2 (83.0–87.2)	3170	90.0 (87.4–91.6)	3169	89.2 (86.8–91.1)
8. Would buy fresh vegetables from Ebola survivor shopkeeper	447	32.0 (26.7–37.9)	1462	70.5 (67.0–73.8)	2934	83.0 (80.3–85.3)	2974	83.5 (80.8–85.9)
9. Ebola survivor student does not put class at risk of Ebola	452	32.8 (25.8–40.7)	1488	71.6 (67.4–75.6)	2541	71.9 (67.5–75.9)	2504	70.4 (66.5–74.0)
**Acceptance of safe burial practices**								
10. Would avoid touching or washing a corpse^f^	ND	ND	1873	90.2 (87.2–92.6)	3362	95.0 (93.9–96.0)	3415	95.9 (94.8–96.8)
11. Would wait for the Ebola burial team to bury the body^f^	ND	ND	1787	86.0 (82.4–90.0)	3404	96.2 (95.0–97.2)	3402	95.5 (94.3–96.5)
12. Would accept safe alternatives to traditional burial rituals^f^	ND	ND	1334	64.3 (59.2–69.0)	3049	86.3 (83.1–89.0)	2823	79.5 (75.6–83.0)
**Self-reported prevention practices**								
13. Uptake of any Ebola prevention practice	1344	95.1 (92.2–97.0)	2022	97.2 (95.7–98.2)	3439	97.3 (96.2–98.0)	3455	97.3 (96.3–97.9)
14. Wash hands with soap and water more often	917	65.8 (59.3–71.7)	1701	81.5 (78.2–84.5)	2790	78.8 (75.7–81.7)	3056	88.5 (85.9–90.6)
15. Avoid physical contact with suspected Ebola patients	498	35.3 (24.1–48.4)	737	35.3 (31.5–39.4)	1538	43.4 (39.5–47.5)	1122	32.5 (28.8–36.3)
16. Avoid burials that involve contact with a corpse^f^	ND	ND	569	27.3 (23.0–32.0)	1673	47.3 (42.9–51.7)	1700	49.2 (45.0–53.4)

CI: confidence interval; ND: not determined.

^a^ The total number of valid responses in the August 2014 survey ranged from 1371 to 1409; missing values accounted for less than  3% of all responses.

^b^ Percentages are of the total number of survey participants.

^c^ The total number of valid responses in the October 2014 survey ranged from 2070 to 2086; missing values accounted for less than  1% of all responses.

^d^ The total number of valid responses in the December 2014 survey ranged from 3534 to 3540; missing values accounted for less than  1% of all responses.

^e^ The total number of valid responses in the July 2015 survey ranged from 3455 to 3563; missing values accounted for less than  4% of all responses.

^f^ Item not included in the first survey in August 2014.

**Table 4:** Ebola knowledge, attitudes and prevention practices before and after the outbreak peak, Sierra Leone, 2014–2015

Ebola knowledge, attitude or prevention practice	Surveys before the outbreak peak^a^				Surveys after the outbreak peak^b^			Odds of respondents giving the desired response after the outbreak peak compared with before^c^
	No. respondents	No. giving a positive response	Percentage giving a positive response (95% CI)		No. respondents	No. giving a positive response	Percentage giving a positive response (95% CI)	aOR (95% CI)
**Knowledge**								
1. Ebola is preventable by avoiding contact with a corpse	3471	3141	90.5 (87.3–92.9)		7099	6741	95.0 (93.9–95.9)	2.1 (1.4–3.0)
2. Early medical care of Ebola increases the chance of survival	3466	3192	92.1 (90.3–93.6)		7097	6791	95.7 (94.9–96.4)	2.4 (1.8–3.2)
3. Early medical care of Ebola reduces household transmission	3483	3226	92.6 (90.7–94.2)		7097	6552	92.3 (91.0–93.4)	1.0 (0.8–1.4)
**Misconception**								
4. Bathing with salt and hot water prevents Ebola	3451	1288	37.3 (34.7–40.1)		7088	1651	23.3 (20.8–26.0)	0.4 (0.3–0.5)
5. Spiritual healers can successfully treat Ebola	3481	553	15.9 (13.3–18.9)		7100	352	5.0 (4.0–6.1)	0.2 (0.1–0.3)
6. Traditional healers can successfully treat Ebola	3484	146	4.2 (3.4–5.1)		7100	112	1.6 (1.2–2.0)	0.3 (0.2–0.5)
**Social acceptance of survivors**								
7. Would welcome back Ebola survivor into the community	3474	2084	60.0 (51.5–67.9)		7089	6339	89.4 (87.8–90.8)	6.0 (3.9–9.1)
8. Would buy fresh vegetables from Ebola survivor shopkeeper	3468	1909	55.0 (49.1–60.8)		7097	5908	83.2 (81.4–85.0)	4.5 (3.4–5.9)
9. Ebola survivor student does not put class at risk of Ebola	3454	1940	56.2 (50.0–62.1)		7094	5045	71.1 (68.2–73.8)	2.1 (1.5–2.9)
**Acceptance of safe burial practices**								
10. Would avoid touching or washing a corpse^d^	2076	1873	90.2 (87.2–92.6)		7098	6777	95.5 (94.7–96.2)	2.3 (1.6–3.3)
11. Would wait for the Ebola burial team to bury the body^d^	2078	1787	86.0 (82.4–88.9)		7100	6806	95.9 (95.0–96.6)	4.4 (3.2–6.0)
12. Would accept safe alternatives to traditional burial rituals^d^	2076	1334	64.3 (59.2–69.0)		7084	5872	82.9 (80.3–85.2)	3.9 (2.8–5.3)
**Self-reported prevention practices**								
13. Uptake of any Ebola prevention practice	3493	3366	96.4 (95.0–97.4)		7087	6894	97.3 (96.7–97.8)	1.5 (0.9–2.2)
14. Wash hands with soap and water more often	3480	2618	75.2 (71.5–78.6)		6995	5846	83.6 (81.5–85.5)	1.9 (1.4–2.5)
15. Avoid physical contact with suspected Ebola patients	3495	1235	35.3 (30.0–41.0)		6995	2660	38.0 (35.2–40.9)	1.3 (1.1–1.7)
16. Avoid burials that involve contact with a corpse^d^	2086	569	27.3 (23.0–32.0)		6995	3373	48.2 (45.2–51.3)	3.1 (2.4–4.2)

CI: confidence interval; aOR: adjusted odds ratio.

^a^ Two surveys were conducted before the outbreak peak, in August and October 2014.

^b^ Two surveys were conducted after the outbreak peak, in December 2014 and July 2015.

^c^ The adjusted odds ratio was derived using a multivariable model adjusted for the regional Ebola transmission level, sex, age, education and religion.

^d^ As this item was introduced in the second survey in October 2014, numbers for the period before the outbreak peak were derived from the October 2014 survey alone.

The proportion of respondents with knowledge that Ebola virus disease is preventable by avoiding contact with corpses increased from 90.5% (3141/3471) to 95.0% (6741/7099; adjusted odds ratio, aOR: 2.1; 95% confidence interval, CI: 1.4–3.0) from before to after the peak and the proportion with the misconception that spiritual healers can successfully treat Ebola decreased from 15.9% (553/3481) to 5.0% (352/7100; aOR: 0.2; 95% CI: 0.1–0.3). The proportion willing to welcome back Ebola survivors into the community increased from 60.0% (2084/3474) to 89.4% (6339/7089; aOR: 6.0; 95% CI: 3.9–9.1) and the proportion who accepted safe alternatives to traditional burials increased from 64.3% (1334/2076) to 82.9% (5872/7084; aOR: 3.9; 95% CI: 2.8–5.3). The proportion who self-reported handwashing with soap increased from 75.2% (2618/3480) to 83.6% (5846/6995; aOR: 1.9; 95% CI: 1.4–2.5) and the proportion who self-reported avoidance of unsafe traditional burials increased from 27.3% (569/2086) to 48.2% (3373/6995; aOR: 3.1; 95% CI: 2.4–4.2).

An analysis of the combined effect of temporal and geographical interactions found that there was a significant interaction for only: (i) the intention to wait for the Ebola burial team if a family member died at home; and (ii) the self-reported avoidance of physical contact with suspected Ebola patients (Table 5). The improvements in the intention to wait for a burial team and in self-reported avoidance of physical contact with patients were greater in high-transmission than low-transmission regions. The likelihood that a respondent would express an intention to wait for a burial team after the outbreak peak compared with before the peak was around three times greater in high-transmission (aOR: 6.2; 95% CI: 4.2–9.1) than low-transmission (aOR: 2.3; 95% CI: 1.4–3.8) regions. Similarly, the likelihood that a respondent would avoid physical contact with suspected Ebola patients was significantly higher after than before the outbreak peak in high-transmission (aOR: 1.9; 95% CI: 1.4–2.5) but not low-transmission (aOR: 0.8; 95% CI: 0.6–1.2) regions.

**Table 5:** Effect of Ebola disease transmission level and survey timing on intention to wait for burial teams and to avoid physical contact with suspected patients, Sierra Leone, 2014–2015

Interaction between transmission level and survey timing	Coefficients used to calculate odds^a^	OR (95% CI)	
		Intention to wait for burial team if family member died	Self-reported prevention practice of avoiding physical contact with suspected Ebola patients
After the outbreak peak versus before the peak in high-transmission regions	exp (*β*1)	6.2 (4.2–9.1)	1.9 (1.4–2.5)
After the outbreak peak versus before the peak in low-transmission regions	exp (*β*1 + *β*3)	2.3 (1.4–3.8)	0.8 (0.6–1.2)
Low- versus high-transmission regions before the outbreak peak	exp (*β*2)	4.1 (2.6–6.5)	3.6 (2.4–5.2)
Low- versus high-transmission regions after the outbreak peak	exp (*β*2 + *β*3)	1.5 (1.0–2.3)	1.5 (1.2–2.0)
After the peak in low-transmission regions versus before the peak in high-transmission regions	exp (*β*1 + *β*2 + *β*3)	9.6 (6.1–15.2)	2.9 (2.1–4.0)

CI: confidence interval; OR: odds ratio.

^a^ The log odds of a specific knowledge, attitude or prevention practice in the multilevel logistic regression model = *β0* + *β1* (stage of outbreak) + *β2* (region) + *β3* (stage of outbreak × region interaction) + *β4* (education) + *β5* (sex) + *β6* (age) + *β7* (religion) + cluster random intercept.

## Discussion

Our findings in the four surveys show that nearly all Ebola knowledge, attitude and practice outcomes improved during the 2014 to 2015 disease outbreak in Sierra Leone. Notably, the proportion of survey respondents who expressed willingness to wait for a safe burial team and to avoid physical contact with suspected patients increased much more in high-transmission regions, where social mobilization efforts were intensified, than in low-transmission regions. However, before the outbreak peak, the likelihood of intending to wait for a burial team was four time greater in low-transmission than high-transmission regions (data available from the corresponding author). Many Ebola cases may have been averted in low-transmission regions as a result. However, as the outbreak progressed and social mobilization activities were intensified, there was a greater change in behaviour in high-transmission regions. Consequently, from before to after the outbreak peak there was a sixfold increase in the proportion of respondents willing to wait for a burial team in high-transmission regions versus a twofold increase in low-transmission regions. Similarly, there was a twofold increase in the proportion avoiding physical contact with suspected Ebola patients in high-transmission regions versus no change in low-transmission regions. A previous study found that the adoption of Ebola prevention practices in Sierra Leone was strongly associated with greater exposure to information on Ebola virus disease.^25^ Hence, together with earlier evidence,^9^,^25^,^26^ our results suggest that social mobilization contributed to controlling the outbreak in high-transmission regions.

Originally, we planned to carry out monthly surveys from August 2014 until the end of the outbreak to observe month-to-month trends in Ebola knowledge, attitudes and practices. However, our experience with the first survey and the prolongation of the outbreak led us to conclude that this was impractical. To ensure data collection was completed within 7 to 10 days, on average, each survey involved about 100 data collectors, 20 team supervisors and 4 regional supervisors. Careful planning was needed to address the complexities of deploying survey teams during an evolving outbreak, particularly to ensure their safety and security. As a result, we opted for bimonthly surveys; hence, the second survey took place in October 2014 and the third, in December. As we observed that improvements in knowledge, attitudes and practices were plateauing after the third survey in December, we waited until the outbreak was nearing its end before conducting the fourth survey. This survey timing enabled us to capture important snapshots of population trends at different stages of the outbreak. Within a few days of each round of data collection, we presented preliminary results to all stakeholders involved in the national response to the Ebola outbreak and highlighted actionable recommendations. It was particularly important that decision-makers responsible for continuously guiding communication and social mobilization strategies were made aware of the preliminary results as soon as possible.^27^

Since WHO declared the West Africa outbreak over in 2016, three further Ebola outbreaks have occurred in the Democratic Republic of the Congo.^28^ In fact, WHO declared the 2018 to 2019 outbreak in North Kivu province a public health emergency of international concern.^29^ Experience with outbreaks in the Democratic Republic of the Congo and West Africa highlighted the recurring challenge of gaining and sustaining community support for the prolonged modification of care-seeking behaviour and traditional burial rituals. An underlying mistrust of the authorities is a common barrier to gaining community support for disease response efforts. In a 2018 survey conducted in North Kivu, for example, only one third of respondents expressed trust in local authorities (mistrust has been associated with not adhering to Ebola prevention practices and not accepting Ebola vaccines).^30^ In Sierra Leone, over 90% of respondents in a survey carried out in July 2015 expressed confidence that the health-care system could treat suspected Ebola cases, though that survey reflected attitudes in the period when the outbreak was waning.^31^

Although our surveys focused on community-level drivers of behaviour, any intervention aimed at increasing Ebola prevention practices must be coordinated with other actions, such as ensuring the timely availability of ambulances and burial services. For instance, delays in responding to death notifications may have caused frustration in the community, which could ultimately have undermined trust in the health services being promoted to the population. To maintain public confidence, it is critical that service delivery is responsive to the level of demand generated in the community by social mobilization.

Our study had several limitations. Survey respondents may have felt it socially desirable to provide responses that matched the messages received through social mobilization efforts. However, we believe their responses probably reflected true knowledge of recommended practices. Second, in the final stage of sampling, systematic sampling might not have produced a truly random selection of households and individuals to interview, particularly because of the difficulty of systematically selecting households in urban slum areas. Nevertheless, the demographic characteristics of our sample were similar to those documented in the latest Demographic and Health Survey in Sierra Leone,^32^ except that respondents with some education were over-represented in our sample. Finally, some differences between or across geographical regions could not be accounted for by studying Ebola cases alone. For example, the larger increase in the proportion of respondents willing to wait for a burial team and to avoid unsafe burial practices in high-transmission regions compared with low-transmission regions may have been influenced by more intensive social mobilization (an extrinsic factor) or by more frequent observation of Ebola patients and their deaths in the community (an intrinsic factor). We were not able to distinguish the effect of social mobilization efforts and lived experiences on improvements in knowledge, attitudes and self-reported practices from our survey data.

Here, we have demonstrated that it is feasible to rapidly conduct serial, community-based surveys of changes in the population’s knowledge, attitudes and practices during an Ebola outbreak and that these surveys can be used to inform response strategies in real time. The marked increase in respondents’ willingness to wait for a safe burial team and to avoid physical contact with suspected Ebola patients in high-transmission regions in Sierra Leone may have been due to experiencing a death in the family or community. However, there is evidence that social mobilization probably contributed to behavioural change and, thereby, helped contain the outbreak.^9^ Social mobilization that targets behaviour and helps translate knowledge of Ebola into prevention practices should be a national priority during Ebola outbreaks, particularly in high-transmission areas. Countries experiencing an Ebola outbreak could consider adopting a similar survey method with standardized outcome measures to assess changes in the population’s knowledge, attitudes and prevention practices.
